# Altered topological properties of functional brain networks in patients with first episode, late-life depression before and after antidepressant treatment

**DOI:** 10.3389/fnagi.2023.1107320

**Published:** 2023-03-06

**Authors:** Chaomeng Liu, Li Li, Weigang Pan, Dandi Zhu, Siyuan Lian, Yi Liu, Li Ren, Peixian Mao, Yanping Ren, Xin Ma

**Affiliations:** ^1^Beijing Key Laboratory of Mental Disorders, National Clinical Research Center for Mental Disorders and National Center for Mental Disorders, Beijing Anding Hospital, Capital Medical University, Beijing, China; ^2^Advanced Innovation Center for Human Brain Protection, Capital Medical University, Beijing, China

**Keywords:** late-life depression, topological properties, functional brain network, antidepressant therapy, resting-state fMRI

## Abstract

**Objectives:**

To preliminarily explore the functional activity and information integration of the brains under resting state based on graph theory in patients with first-episode, late-life depression (LLD) before and after antidepressant treatment.

**Methods:**

A total of 50 patients with first-episode LLD and 40 non-depressed controls (NCs) were recruited for the present research. Participants underwent the RBANS test, the 17-item Hamilton depression rating scale (HAMD-17) test, and resting-state functional MRI scans (rs-fMRI). The RBANS test consists of 12 sub-tests that contribute to a total score and index scores across the five domains: immediate memory, visuospatial/constructional, language, attention, and delayed memory. Escitalopram or sertraline was adopted for treating depression, and the dosage of the drug was adjusted by the experienced psychiatrists. Of the 50 LLD patients, 27 cases who completed 6-month follow-ups and 27 NCs matched with age, sex, and education level were included for the final statistical analysis.

**Results:**

There were significant differences in RBANS total score, immediate memory, visuospatial/constructional, language, attention, and delayed memory between LLD baseline group and NCs group (*P* < 0.05). Considering the global attribute indicators, the clustering coefficient of global indicators was lower in the LLD baseline group than in the NCs group, and the small-world attribute of functional brain networks existed in all three groups. The degree centrality and node efficiency of some brains were lower in the LLD baseline group than in the NCs group. After 6 months of antidepressant therapy, the scores of HAMD-17, immediate memory, language, and delayed memory in the LLD follow-up group were higher than those in the LLD baseline group. Compared with the LLD baseline group, the degree centrality and node efficiency of some brains in the cognitive control network were decreased in the LLD follow-up group.

**Conclusions:**

The ability to integrate and divide labor of functional brain networks declines in LLD patients and linked with the depression severity. After the relief of depressive symptoms, the small-world attribute of functional brain networks in LLD patients persists. However, the information transmission efficiency and centrality of some brain regions continue to decline over time, perhaps related to their progressive cognitive impairment.

## Introduction

Depression after the age of 60–65 years is known as late-life depression (LLD), affecting 4–10% of elderly individuals (Sjöberg et al., 2017; Li et al., 2020). LLD is often related to aging-associated neurodegeneration, cognitive impairment, or somatic complaints compared to the major depressive disorder (MDD) observed in the younger generation, increasing the risk for Alzheimer's disease (AD), disability, and mortality (Manning et al., 2019). Approximately 30–50% of patients with LLD still have cognitive impairment after the relief of depressive symptoms (Kim and Han, 2021). A neuropsychological study found that executive function impairment still exists among patients with LLD in the remission stage and has a potential predictive effect on memory impairment, suggesting that cognitive impairment, especially executive function impairment, may be a characteristic pathological change in patients with LLD and not associated with the remission of depressive symptoms (Liao et al., 2017). The cognitive impairment of LLD patients, such as executive function, episodic memory, language, and social ability, may be associated with cerebrovascular disease, hippocampal atrophy, changes in corticosteroids, and immune inflammation (Invernizzi et al., 2021). However, the underlying neuropathological mechanisms of cognitive impairment remain unclear.

Modern brain neuroimaging techniques have recently developed rapidly. Functional magnetic resonance imaging (fMRI) is commonly used owing to its non-invasiveness, non-requirement of radioactive tracer exposure, and providing new insights into the pathophysiology of depression. Resting-state fMRI (rs-fMRI) is a feasible and widely-accepted method, as depicted in an earlier study (Biswal et al., 1995). The study first reported that the spontaneous low frequency (0.01–0.08 Hz) fluctuations were closely associated with intrinsic brain activity and physiology. Many rs-fMRI studies have demonstrated an abnormal activation of the frontal lobe-limbic brain region in patients with LLD. We recently discovered that in drug-naive, first-episode patients with LLD, a lack of activation of the bilateral superior frontal gyrus, orbital part, and bilateral anterior cingulate cortex (ACC) was associated with attention deficit (Liu et al., 2022). Furthermore, a cross-sectional study found abnormal frontal lobe-limbic system activation and negative emotion attention bias in patients with remitted LLD (rLLD). They also proposed that dysfunctions in the appropriate temporal-cerebellum neural circuit may contribute to the similarities seen in rLLD and amnesiac mild cognitive impairment (MCI) conversion to AD (Chen et al., 2016).

In summary, LLD cognitive impairment may be associated with abnormal activity in areas related to the frontal-parietal lobe and limbic system. Kumar and Cook proposed that structural alterations, such as frontal and subcortical volumetric changes, adhere to the functional alterations seen in LLD (Kumar and Cook, 2002). Depression has increasingly been postulated as an alteration of whole-brain connectome organization, which could serve as a specific diagnostic neuromarker and therapeutic evaluation tool (Gong and He, 2015; Kaiser et al., 2015). Given that the hypothesized disruption in functional connectivity and its implications for behavior, methods to ascertain the topological organization of these networks would be of particular utility in understanding LLD. However, the most of previous studies on graph theoretical analyses in LLD had a cross-sectional design, and their findings may have been limited because of confounding variables like antidepressants and recurrence, which make it impossible to fully comprehend the functional activity and information integration of the whole brain over time of patients with LLD.

Graph theory employs mathematical methods to abstract complex brain networks into simple geometric figures comprised of nodes and edges, where nodes can be electroencephalogram electrodes and magnetoencephalography channels or the region of interest (ROI) defined on the commonly used brain structure and function template, and the edge primarily refers to functional or structural connections between the nodes (Luo et al., 2021). The topology of the human brain can be divided into two categories: global and node attributes. Clustering coefficient (Cc), Characteristic path length (Lp), Small world index (Sigma), and Global efficiency (Eg) are the most commonly used global indicators, while degree centrality (DC) and node efficiency (Ne) are the most commonly used node indicators (Wang et al., 2016). Graph theory analysis can explore the organizational pattern of the brain quantitatively and reveal its information flow characteristics (Fasmer et al., 2018). Cc in the global attribute index represents the degree of aggregation between network nodes and neighboring nodes. The higher the Cc, the more developed the functional division of labor in the network and the greater the ability to transmit information. Lp is the average length of the shortest path between all node pairs in the network, and it reflects the network's overall functional integration and global information transmission capabilities. The lower the Lp, the greater the functional integration and information transmission capability. The small world network has a higher Cc and a shorter Lp, as well as high local and overall efficiency, which is also the attribute characteristic of the normal brain, reflecting the balance between the division of labor and integration abilities of the whole brain network and ensuring the efficiency of information transmission (Achard and Bullmore, 2007). The symbol Sigma (σ) can be used to represent this feature of the small world network.


$$\begin{array}{l} Sigma\left(\sigma \right)=\frac{\gamma }{\lambda }=\frac{C/C_{rand}}{L/L_{rand}} \end{array}$$

In the above formula, C and L denote the Cc and Lp, respectively. C_rand_ and L_rand_ represent, respectively, the average Cc and average Lp of random networks with the same number of nodes and edges. The standardized Cc and Lp are denoted by the symbols gamma (γ) and lambda (λ). In general, the network is considered to have small-world properties when σ > 1 (Kawai et al., 2019). DC refers to the degree of each node in the network in the node attribute index, and the higher the value, the more likely the node is to become a hub in the network. Ne, like Eg, is defined as the reciprocal of the average distance of the shortest path length between any two nodes *via* a node. The greater the value, the faster the data transmission (Lim et al., 2013; Farahani et al., 2019).

Previous researches investigated the small-world properties of the LLD brain network using large-scale graph theory analysis. For example, Ajilore et al. (2014) found that the small-world attribute of functional brain network in patients with LLD patients still existed compared with the control group; however, Eg was significantly reduced, and this may reflect the underlying neuroanatomical vulnerabilities of LLD (Ajilore et al., 2014). According to Wang et al. (2016), the topological organization of functional brain networks of LLD is disrupted, which may contribute to the disturbance in cognitive impairment in LLD patients (Wang et al., 2016). However, the changes in topological properties of functional brain networks among patients with LLD after antidepressant therapy were uncertain.

In the past few decades, Selective serotonin reuptake inhibitors (SSRIs) have been regarded as the first-line drugs for the treatment of LLD, such as fluoxetine, paroxetine, sertraline, fluvoxamine, citalopram, and escitalopram (Beyer and Johnson, 2018). SSRIs have fewer adverse reactions and better tolerance compared with other antidepressants, of which escitalopram and sertraline have the highest ratings (Alexopoulos et al., 2001). This study aimed to comprehensively understand the effect of antidepressant drugs on the functional activity and information integration of the brains in patients with LLD using rs-fMRI and graph theory analysis. Here, we concentrate on the global attribute indicators mainly since it might, to some extent, represent the level of cognitive impairment in LLD patients. We assumed that cognitive impairment in patients with first-episode, drug-naive LLD persisted after the relief of depressive symptoms, and the cognitive impairment may be related to the changes in some global attribute indicators of the functional brain network.

## Materials and methods

### Participants

Patients with LLD were recruited from the outpatient services of Beijing Anding Hospital, Capital Medical University (Beijing, China). All participants were aged between 60 and 75 years, right-handed, with the first episode and no previous treatment with psychotropic drugs, with an education level of more than 6 years, and confirmed by a certified geriatric psychiatrist through Axis I major depressive episode according to the DSM-IV or DSM-V through the diagnostic interview (Mendes-Silva et al., 2021). Based on the above diagnostic criteria, five of the core symptoms triggered by various life events or no obvious triggers must be present for at least 2 weeks for a diagnosis of LLD to be fulfilled; one symptom must be depressed mood or loss of interest/enjoyment in everyday activities (anhedonia). The conditions must be met for the symptoms to have a considerable effect on social and/or occupational functioning (Meunier et al., 2009). In addition, no cases were diagnosed with additional Axis I major psychiatric disorders, except for anxiety disorders. Patients enrolled in the study had at least a score of 17 evaluated using the 17-item Hamilton depression rating scale (HAMD-17) (Pan et al., 2022). Age, sex, and education level-matched healthy controls (HCs) who had no history of mental disorders were recruited through internet advertisements. Additionally, all the participants were requested to achieve the minimum score of 24 on the Mini-Mental State Examination scale (excluding the presence of dementia) (Bohr et al., 2012). The exclusion criteria were as follows: major neurocognitive decline and head trauma history, Parkinson's disease, stroke, and critical cardiovascular, respiratory, immune, and other systemic diseases. Informed consent was taken from all participants. The study protocols were approved by the institutional review board of Beijing Anding Hospital.

### Antidepressant therapy

**Figure 1:**
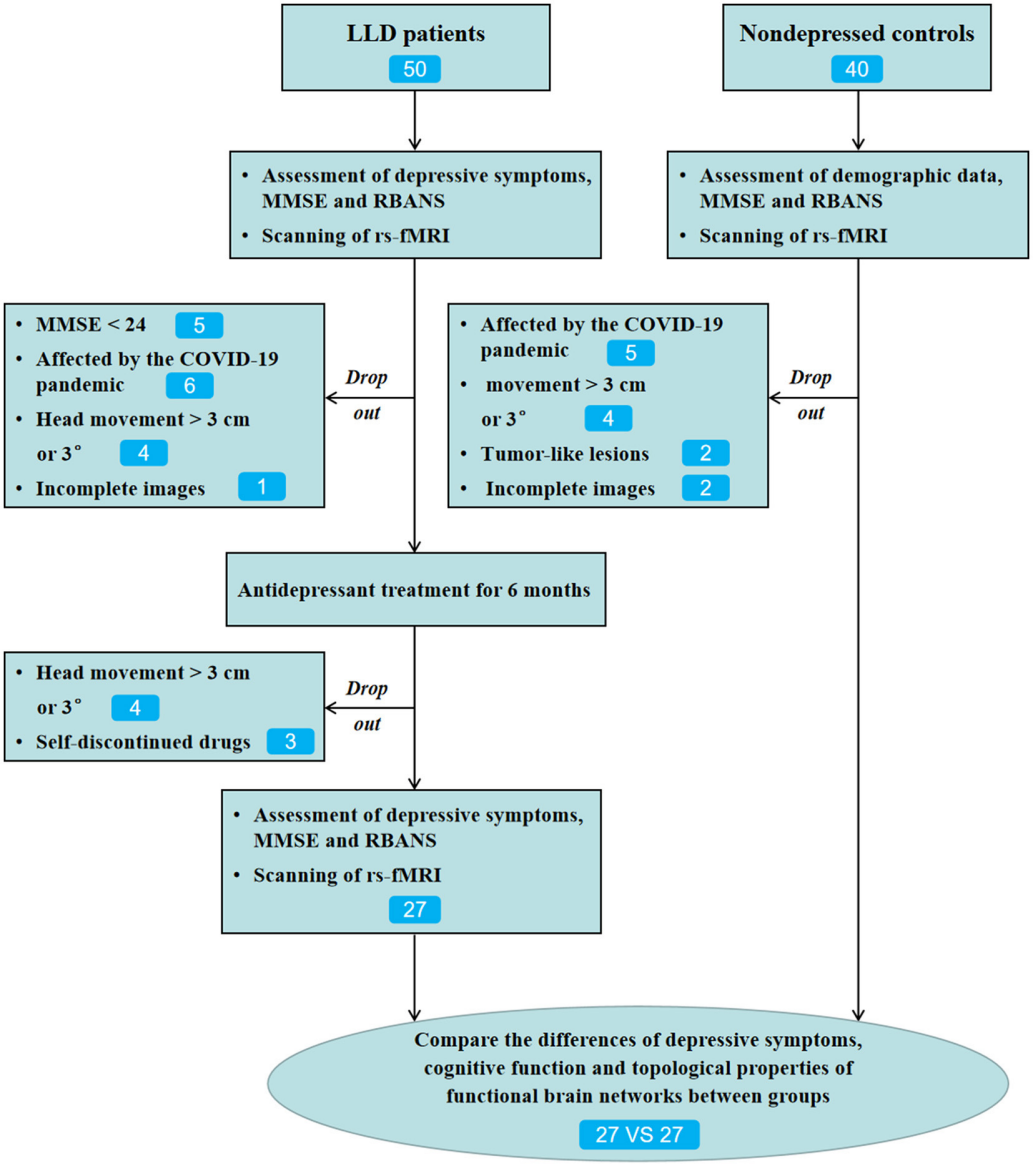
Flow diagram of this study.

At baseline, a total of 50 patients with LLD who met the inclusion criteria were included. Therefore, in this study, escitalopram (5–15 mg/day) or sertraline (25–150 mg/day) was used for treating depression, and the dosages of the drugs were within the safe and effective ranges, and there was no off-label use based on the previous studies (Beyer and Johnson, 2018; Jannini et al., 2022). Patients with severe sleep disturbance, anxiety, or agitation may be treated using a short-acting benzodiazepine (lorazepam 0.5 mg, 0.5 mg-1 mg each time). Of the 50 patients with LLD, 34 completed 6-month follow-ups. Among them, 23 cases left the study due to different reasons (Figure 1). A total of 27 patients with LLD who completed the 6-month follow-up and 27 NCs matched with age, sex, and education level were included for the final statistical analysis.

### Depressive symptoms and cognitive function assessment

No patients received neurostimulation therapy such as electroconvulsive or transcranial magnetic stimulation therapy during the 6-month treatment period. The HAMD-17 was used to assess clinical symptoms at baseline and 6 months after treatment. Response rates were calculated as a 50% reduction in HAMD-17 total scores from baseline to 6 months after treatment.

The current study employed well-trained clinicians to conduct a cognitive assessment using the Repeatable Battery in the Assessment of Neuropsychological Status (RBANS) (Randolph et al., 1998). It includes 12 standardized cognitive tests divided into the following five categories: visuospatial/constructional (line orientation, figure copy); immediate memory (story memory, list learning); attention (digit symbol coding, digit span); language (semantic fluency, picture naming); and delayed memory (semantic fluency, picture naming; list recognition, list recall, figure recall, story recall). A higher RBANS score indicates better cognitive performance. Previous research has shown that RBANS is a useful screener for assessing cognitive impairments in patients with psychiatric disorders (Bohr et al., 2012). The LLD group was assessed for cognitive function at baseline and 6 months after treatment. HCs group completed the cognition test once when entering the trial.

### Rs-fMRI protocol and data analysis

All participants were scanned using the Siemens 3T scanner (Siemens, Erlangen, Germany), and the head was tightly fastened using foam pads and straps to avoid motion. The present study initially captured T1 images (preliminarily excluding intracranial organic diseases, such as tumor-like lesions or absence of the disease), and then the rs-fMRI was obtained (~7 min). When collecting resting-state fMRI data, each participant was asked to relax with eyes closed, lie still, and keep awake. The T1 images was gathered using the T1-weighted sagittal 3D magnetization-prepared rapid gradient echo sequence: echo time (TE) = 1.85 ms; repetition time (TR) = 2530 ms; field of view (FOV) = 256 × 256 mm^2^; flip angle (FA) = 9°; voxel size = 1.0 × 1.0 × 1.0 mm^3^; thickness = 1.0 mm, and matrix size = 256 × 256. Then, images were gathered in resting-state axially with the echo-planar imaging (EPI) sequence under the following parameters, TE = 30 ms; T*R* = 2000 ms; FOV = 256 × 256 mm^2^; FA = 90°; axial slices = 33; matrix size = 64 × 64; slice thickness = 3.5 mm, voxel size = 3.1 × 3.1 × 3.5 mm^3^; and a total of 200 time points (Liu et al., 2022).

MRIcro software was used to classify and analyze functional images (www.MRIcro.com). RESTplus V1.2 (http://www.restfmri.net) toolbox based on the MATLAB R2018b platform was used to preprocess the data (Liu et al., 2022). Initially, form transformation (DICOM to NIFTI) was performed, followed by excluding the first 10 functional volumes, slice timing, head motion correction, Montreal Neurological Institute space normalization (using T1 image unified segmentation), and re-sampling data at 3 × 3 × 3 mm^3^ resolution, smoothing (full-width Gaussian kernel = 6 × 6 × 6 mm^3^) (0.01–0.08 Hz). In regressing the head motion effects, the current study used Friston's 24 head-motion parameters as covariates. For white matter, global mean signal, cerebrospinal fluid signal, and head motion, linear regression was used to remove covariates.

### Defining the nodes and edges of the functional brain network

There are numerous methods for partitioning the human brain on a macro and large scale, one of which is the anatomical automatic labeling (AAL) atlas, which is drawn and segmented layer by layer through the central sulcus of the standard brain's T1 image. The AAL116 template divides the cerebral cortex into 116 ROIs, including 90 brain regions and 26 cerebellum regions, all of which are symmetrical (Rolls et al., 2020). The AAL116 template is used in this study to build the functional brain network, which consists of 116 ROIs as nodes. In addition, a functional brain template called Dosenbach 160 was introduced to validate the results of global attribute indicators from the AAL116 template. Dosenbach 160 functionally define the regions of interest from several meta-analyses of fMRI activation studies (Dosenbach et al., 2010).

The edge of the brain network in the AAL116 template is the strength of the connection between any two ROIs. The absolute value of Pearson's correlation coefficient or Partial correlation coefficient is commonly used to calculate connection strength. When the sample size is >50, the partial correlation coefficient is preferable because the absolute value of Pearson's correlation coefficient becomes smaller in this case. Because the sample size of the single group in our study was 27, Pearson's correlation coefficient was used to assess the relationship between the two nodes (Qiao et al., 2022).

### Construction of functional brain networks

Following rs-fMRI image preprocessing, signal sequences of 190-time points in each brain region (i.e., nodes) were extracted (the first 10 time points were removed to obtain stable magnetization signals). Pearson's correlation method was used to generate the function matrix for each subject. Fisher's Z transformation was used to obtain the Z score matrix of 116 × 116 in order to bring the function matrix closer to the normal distribution. Furthermore, because the small world network is sparse, this study set the sparsity range to 0.05–0.5 and the step size to 0.01, resulting in the construction of a weighted brain functional network for each subject (Zhang et al., 2020). The GretnaV2.0.0 software (https://www.nitrc.org/projects/gretna/) was used to calculate the commonly used global and node attribute indicators in the NCs and LLD functional brain networks (including the LLD baseline and follow-up groups).

### Statistical analysis

Statistical analysis was performed using SPSS26.0 software (SPSS, Chicago, IL, USA) and GretnaV2.0.0 toolbox based on the MATLAB R2018b platform. The independent descriptive variables (age) were expressed as mean ± SD, whereas categorical variables (sex) were expressed as counts and percentages. An independent sample *t*-test or paired sample *t*-test was performed for comparing continuous variables, while a comparison of categorical variables was performed using Chi-square or Mann-Whitney U test. Statistical test differences between the LLD and NCs groups demonstrated statistical significance with *p*-values < 0.05. The differences in graph theory indicators, such as Cc, Lp, Sigma, Eg, DC, and Ne were compared in the GretnaV2.0.0 toolbox, with independent sample *t*-test for the comparisons between NCs group and LLD baseline group and paired sample *t*-test for the comparisons between LLD baseline group and LLD follow-up group. The False Discovery Rate (FDR) method was used to correct the results of multiple comparisons. To maintain the balance between groups, confounding covariates such as age, sex, and education level were regressed. Based on the MATLAB R2018b platform, the brain regions with significant differences in topology node attributes between groups are presented using the BrainNetViewer1.7 toolkit (https://www.nitrc.org/projects/bnv/). Correlation coefficients between HAMD-17 scores, RBANS scores, and global attribute indicators were calculated using the Pearson test. Bonferroni correction was applied to reduce the type I error due to multiple testing (Holm, 1979). The significance level was set to *p* < 0.05, two-tailed.

## Results

### Comparisons of general data, depressive symptoms, and cognitive function

Nineteen of the 27 patients with LLD who completed the follow-up were given escitalopram oxalate, and eight were given sertraline hydrochloride. The LLD baseline group's total RBANS score and factor RBANS scores (including immediate memory, visuospatial/constructional, language, attention, and delayed memory) were significantly lower than those in the matched NCs group (see Table 1).

**Table 1:** Demographics and neuropsychologic data between the LLD baseline group and NCs group.

**Characteristic**	**LLD baseline group**	**NCs group**	***T/X^2^*values**	***P-*values**
	**(*****n** =* **27)**	**(*****n** =* **27)**		
Sex (male/female)	7/20	13/14	2.859	0.091
Age (years)	67.00 ± 4.76	65.71 ± 4.18	1.064	0.292
Education (years)	9.37 ± 2.87	10.81 ± 3.15	−1.765	0.084
RBANS total score	97.48 ± 11.49	118.48 ± 14.99	−5.777	< 0.001^*^
Immediate memory	95.52 ± 12.96	111.81 ± 14.56	−4.345	< 0.001^*^
Visuospatial/Constructional	98.59 ± 10.31	107.78 ± 8.79	−3.522	0.001^*^
Language	96.41 ± 10.89	106.37 ± 6.12	−4.143	< 0.001^*^
Attention	111.63 ± 12.36	122.85 ± 8.44	−3.895	< 0.001^*^
Delayed memory	93.93 ± 10.86	104.93 ± 10.19	−3.838	0.001

RBANS, Repeatable Battery for the Assessment of Neuropsychological Status. ^*^The difference is statistically significant (P < 0.05).

**Figure 2:**
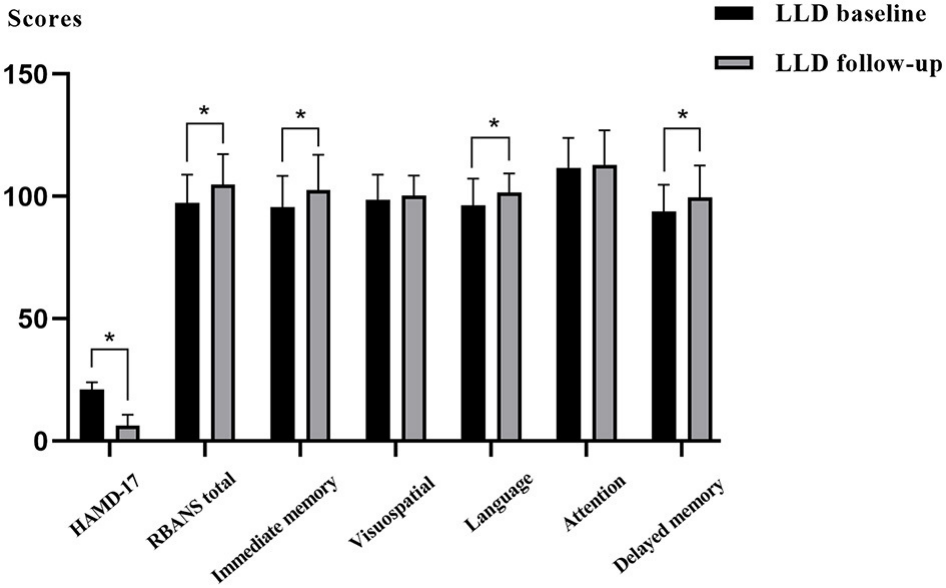
Comparisons of depressive symptoms and cognitive function between the LLD baseline group and LLD follow-up group.

After 6 months of antidepressant therapy, HAMD-17 scores in the LLD follow-up group were lower than those in the LLD baseline group, whereas RBANS, immediate memory, delayed memory, and language scores in the follow-up group were significantly higher than those in the baseline group (*p* < 0.05). Figure 2 and Supplementary Table 1 also showed that there were no significant differences in visuospatial/constructional and attention between the two groups (*p* > 0.05).

### Comparisons of global attribute indicators of functional brain networks

**Figure 3:**
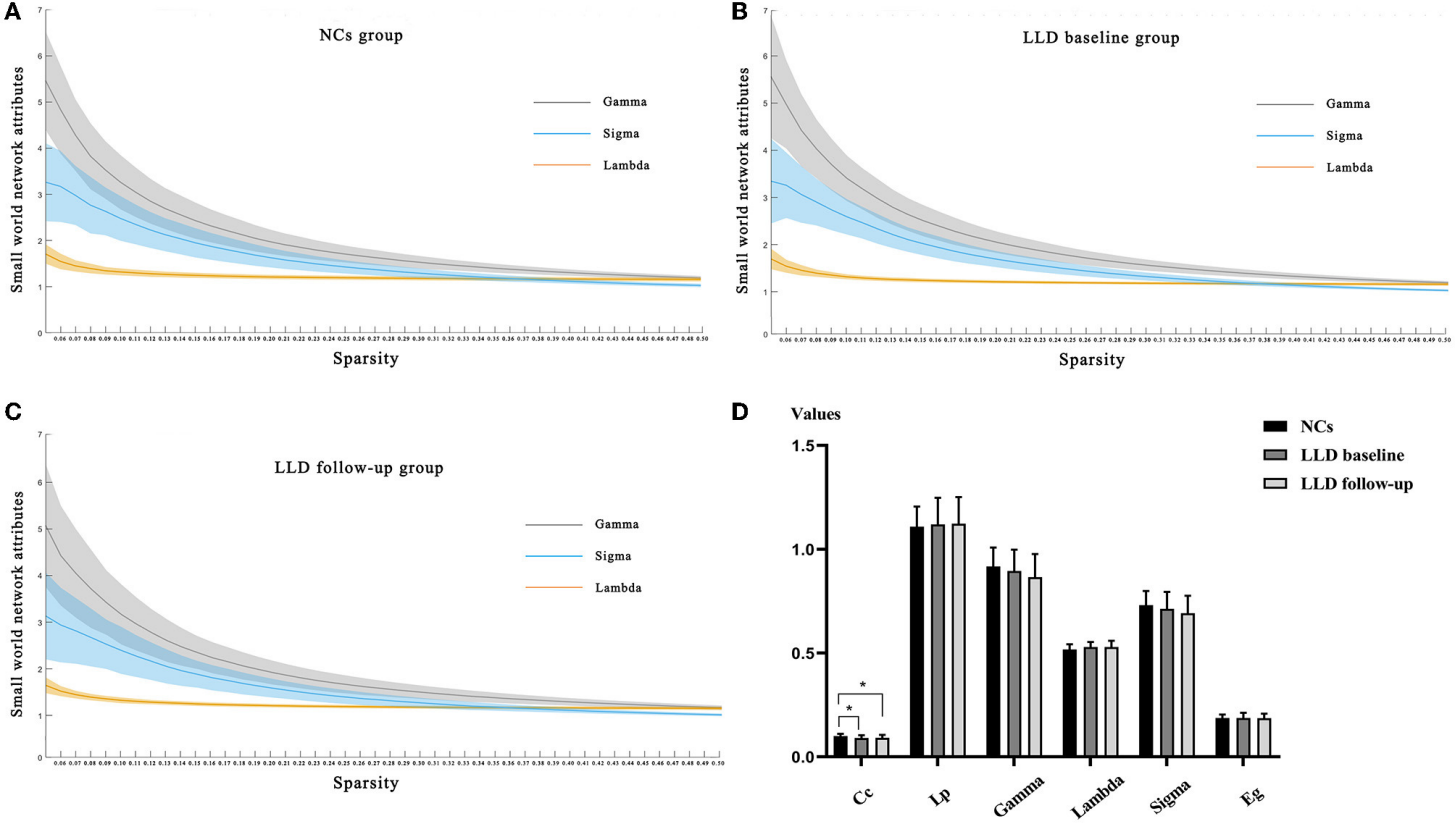
Comparisons of global indicators of functional brain networks from the AAL160 template. **(A)** Small-world attribute indices of functional brain networks along with sparsity in the NCs group; **(B)** Small-world attribute indices of functional brain networks along with sparsity in the LLD baseline group; **(C)** Small-world attribute indices of functional brain networks along with sparsity in the LLD follow-up group; **(D)** Global attribute indicators of functional brain networks in LLD baseline group, LLD follow-up group and NCs group.

The GretnaV2.0.0 toolkit based on the MATLABR2018b platform was used to compare the global attribute indicators of functional brain networks in the LLD baseline, LLD follow-up, and NCs groups, including Cc, Lp, Gamma, Lambda, Sigma, and Eg. The results of Sigma > 1 from the AAL 116 template and the Dosenbach 160 template both indicated that there are small-world attributes in all three groups, as shown in Figures 3A–C and Supplementary Figures 1A–C.

Within the sparsity range of 0.05–0.50, whether from the AAL 116 template or the Dosenbach 160 template, the Cc values of global attribute indicators in the LLD baseline group and LLD follow-up group were lower than those in the NCs group, and the differences were statistically significant (*p* < 0.05). There were no significant differences in Lp, Gamma, Lambda, Sigma, and Eg of global attribute indicators between the three groups (*p* > 0.05) (Figure 3D, Supplementary Figure 1D and Supplementary Tables 2, 3).

### Correlation analysis of HAMD-17 scores, RBANS scores, and global attribute indicators

**Figure 4:**
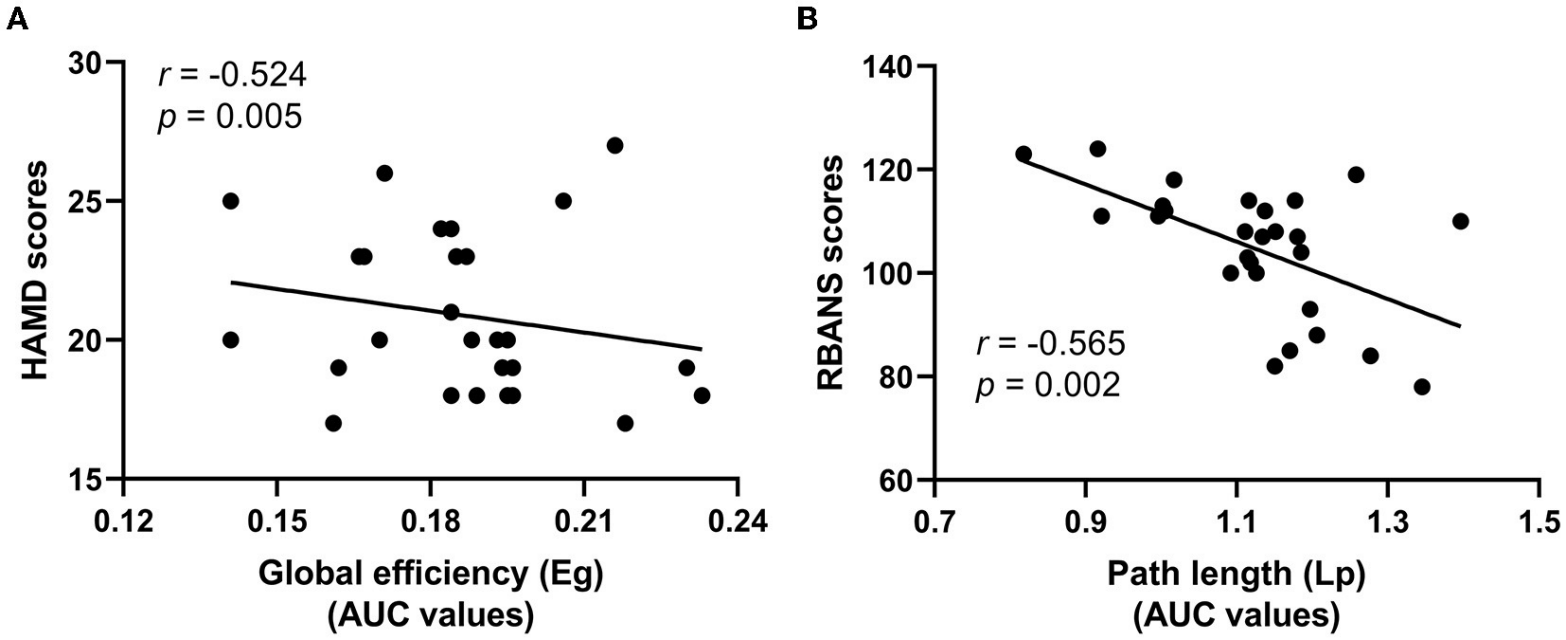
Correlation analysis of HAMD-17 scores, RBANS scores, and global attribute indicators. **(A)** The Cc values of functional brain networks were inversely correlated with the scores of HAMD-17 in the LLD baseline group; **(B)** The Lp values of functional brain networks were inversely correlated with the RBANS scores in the LLD follow-up group.

Linear correlation analysis observed the negatively correlation between the Cc values of functional brain networks and the scores of HAMD-17 in the LLD baseline group (*r* = −0.524, *p* = 0.005, Bonferroni correction, Figure 4A); additionally, the Lp values of functional brain networks were inversely correlated with the RBANS scores in the LLD follow-up group (*r* = −0.565, *p* = 0.002, Bonferroni correction, Figure 4B).

### Comparisons of node attribute indicators of functional brain networks

Based on the MATLABR2018b platform, the GretnaV2.0.0 toolkit was used to compare the node attribute indicators of functional brain network from the AAL 116 template in the LLD baseline, LLD follow-up, and NCs groups, including Ne and DC. The BrainNetViewer1.7 toolkit was adopted to display the brain regions with differences in node attribute indicators between groups.

**Figure 5:**
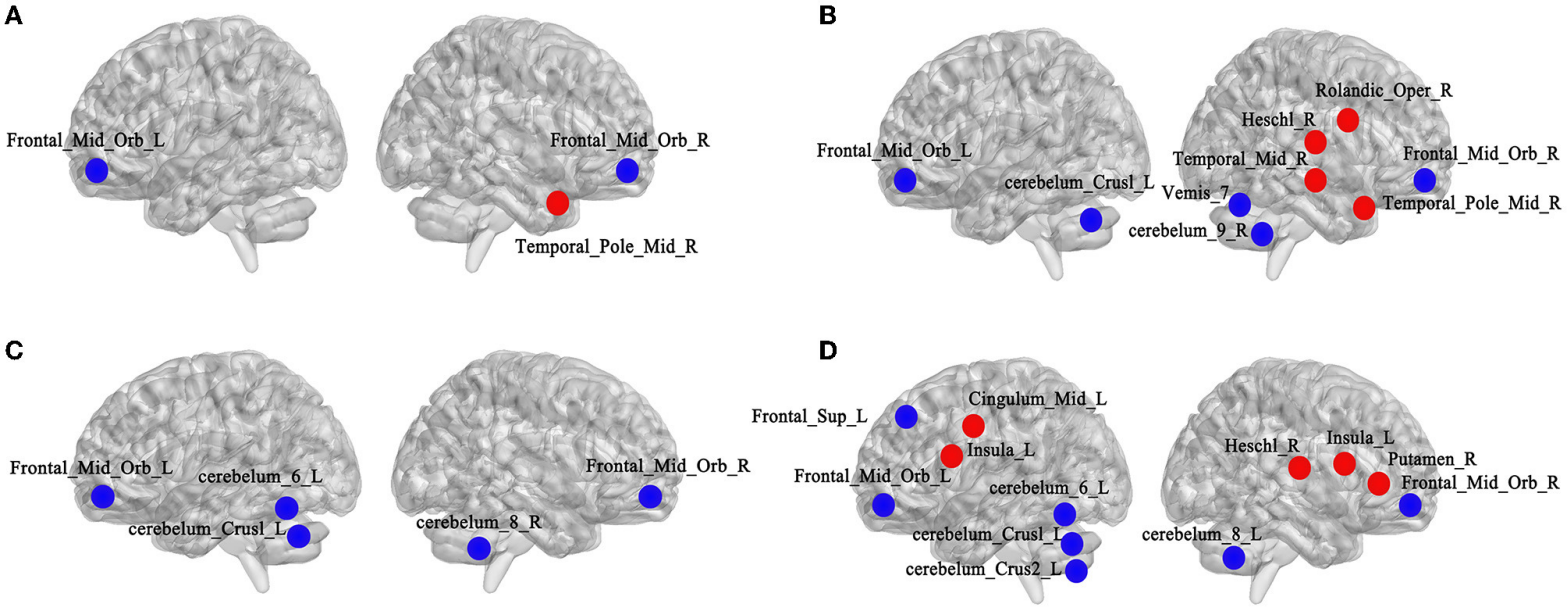
Comparisons of node attribute indicators of functional brain networks from the AAL160 template. **(A)** Altered brain regions of Ne between the NCs and LLD baseline groups (left side is left hemisphere, right side is right hemisphere, red for attribute enhancement, blue for attribute reduction); **(B)** Altered brain regions of DC between the NCs and LLD baseline groups; **(C)** Altered brain regions of Ne between the LLD baseline and LLD follow-up groups; **(D)** Altered brain regions of DC between the LLD baseline and LLD follow-up groups.

In terms of Ne, the Ne values of bilateral middle frontal gyrus, orbital part (Frontal_Mid_Orb) in the LLD groups were lower than those in the NCs group (left: *t* = 2.518, *p* = 0.015; right: *t* = 2.485, *p* = 0.016), and the Ne value of right temporal pole: middle gyrus (Temporal_Pole_Mid_R) was higher in the LLD baseline group than in the NCs group (*t* = 2.908, *p* = 0.005), with a significant difference (*p* < 0.05, FDR correction) as illustrated in Figure 5A.

As for DC, the DC values of bilateral Frontal_Mid_Orb (left: *t* = 2.805, *p* = 0.007; right: *t* = 2.776, *p* = 0.008), cerebelum_Crus1_L (*t* = 2.066, *p* = 0.044), cerebelum_9_R (*t* = 2.185, *p* = 0.033), and Vemis_7 (*t* = 2.561, *p* = 0.013) in the LLD baseline group were lower than those in the NCs group. The DC values of right rolandic operculum (Rolandic_Oper_R) (*t* = 2.168, *p* = 0.035), heschl gyrus (Heschl_R) (*t* = 2.140, *p* = 0.037), middle temporal gyrus (Temporal_Mid_R) (*t* = 2.257, *p* = 0.028), and Temporal_Pole_Mid_R (*t* = 2.023, *p* = 0.048) were higher in the LLD baseline group than in the NCs group, with a significant difference (*p* < 0.05, FDR correction, illustrate Figure 5B).

In terms of Ne, compared with the LLD baseline group, the Ne values of bilateral Frontal_Mid_Orb (left: *t* = 2.319, *p* = 0.029; right: *t* = 2.138, *p* = 0.042), cerebelum_Crus1_L (*t* = 2.234, *p* = 0.034), cerebelum_6_L (*t* = 2.132, *p* = 0.043) and cerebelum_8_R (*t* = 2.190, *p* = 0.038) decreased in the LLD follow-up group, and the difference was statistically significant (*p* < 0.05, FDR correction) as illustrated in Figure 5C.

As for DC, compared with LLD baseline group, the DC values in left superior frontal gyrus, dorsolateral (Frontal_Sup_L) (*t* = 2.264, *p* = 0.032), Frontal_Mid_Orb (left: *t* = 2.315, *p* = 0.029; right: *t* = 2.542, *p* = 0.017), cerebelum_Crus1_L (*t* = 2.838, *p* = 0.009), cerebelum_Crus2_L (*t* = 2.085, *p* = 0.047), cerebelum_6_L (*t* = 3.156, *p* = 0.004), and cerebelum_8_R (*t* = 2.167, *p* = 0.041) decreased in LLD follow-up group. The DC values of bilateral insular (left: *t* = 2.161, *p* = 0.040; right: *t* = 2.117, *p* = 0.044), left median cingulate and paracingulate gyrus (Cingulum_Mid_L) (*t* = 2.418, *p* = 0.023), right lenticular nucleus, putamen (Putamen_R) (*t* = 2.108, *p* = 0.045), and Heschl_R (*t* = 2.169, *p* = 0.039) increased in the LLD follow-up group, with a statistically significant difference (*p* < 0.05, FDR correction) as depicted in Figure 5D.

## Discussion

This study was the first to investigate the effects of antidepressant drugs on the topological properties of functional brain networks in the first-episode, drug-naive patients with LLD. In a relatively long follow-up window, the primary findings were as follows: as for neural cognition, after 6 months of antidepressant therapy, the immediate memory, language, and delayed memory ability of the LLD follow-up group recovered compared with the LLD baseline group; however, no significant difference existed in visuospatial/constructional and attention ability between the two groups with the relief of depressive symptoms. In terms of the topological properties of the functional brain networks, considering the global attribute indicators, the Cc value of global indicators was lower in the LLD baseline group than that in the NCs group. Additionally, after 6 months of antidepressant therapy, the small world attribute of functional brain networks still existed in the first-episode, drug-naive patients with LLD. As to the node attribute indicators, there are some altered brain regions of Ne or DC among the three groups.

### Antidepressant therapy and cognitive functions

After the relief of depressive symptoms, whether the impaired cognitive function of LLD patients would recover accordingly is uncertain. For example, Culang et al. (2009) found that after 8 weeks of citalopram intervention, compared with the baseline, the psychomotor speed and visual-spatial ability of patients with LLD improved greatly, along with the relief of depressive symptoms; however, the impairment of executive function and memory ability persisted (Culang et al., 2009). A community-based large sample follow-up study found that regardless of whether the depressive symptoms were relieved or not, patients with LLD demonstrated a decline in episodic memory and attention function during 3 and 12-month follow-up compared with the non-depressive elderly controls. Moreover, the remission of depressive symptoms did not appear to be necessarily related to the decline of cognitive function (Riddle et al., 2017).

Currently, the effects of antidepressants on cognitive function (positive or negative) are not established. An earlier meta-analysis demonstrated that depression increased the risk of developing AD in elderly individuals, which may be a prodromal manifestation of AD (Mourao et al., 2016). A large retrospective study found that long-term use of antidepressants (≥2 years) could effectively reduce the risk of developing AD in patients with LLD. This could be possible owing to SSRIs antidepressants reducing the burden of AD-related amyloid plaques by modifying the precursor of amyloid protein (Bartels et al., 2020). Conversely, studies have depicted that antidepressant use was associated with an increased risk of dementia, particularly for individuals who start taking antidepressants before the age of 65 years (Moraros et al., 2017). A retrospective cohort study recently demonstrated that neurotoxicity to different types of cells increased the side effects on the nervous system, extrapyramidal reactions, and anticholinergic properties, which may be the underlying mechanism increasing the dementia risk of antidepressants (Kodesh et al., 2019). Moreover, a population-based retrospective case-control analysis has suggested that dementia risk increases with the accumulation of SSRI doses (Lee et al., 2016). The results of previous studies on the beneficial or harmful effects of antidepressants on the cognition of elderly individuals are diverse and have not yet formed a unified understanding.

Our study found that after 6 months of antidepressant therapy, the immediate and delayed memory of patients in the LLD follow-up group was significantly improved compared with the LLD baseline group; however, no significant difference existed in visuospatial/constructional and attention ability between the two groups. Bondareff et al. (2000) conducted a randomized, double-blind study and found similar antidepressant effects of sertraline (50–150 mg/day) and nortriptyline (25–100 mg/day) for 12 weeks; however, sertraline effectively improved memory and daily living abilities of patients with LLD (Bondareff et al., 2000). A previous meta-analysis showed that antidepressants had a positive effect on psychomotor speed and delayed recall in MDD patients, but did not reach statistical significance for cognitive control and executive function (Rosenblat et al., 2015). Our findings are somewhat consistent with this meta-analysis. Several studies have shown that patients with LLD often have severe white matter hyper signal (WMH), which may lead to the damage of the frontal striatal pathway, which is closely related to cognitive function (Delaloye et al., 2010; Feng et al., 2014). Additionally, previous studies have found that the more severe the WMH, the more obvious the impairment of processing speed and executive function (Köhler et al., 2010; Vasudev et al., 2012). After the relief of depressive symptoms, continuous impairment of processing speed and executive functions may be related to the damage of the frontal striatal pathway in patients with LLD. Only patients with LLD were followed up in this study, and the NCs group was not followed up at the end of the 6th month; therefore, the cognitive performance of the LLD follow-up group was not compared with that of the NCs group at baseline.

### Antidepressant therapy and topological attribute index

An earlier study found that with the progressive aggravation of cognitive impairment, disorder degree of brain networks among patients with early and late MCI and AD gradually deepened, and efficiency of information transmission gradually decreased, suggesting that the changes in the topological properties of functional brain networks could reflect the degree of cognitive impairment to some extent (Xiang et al., 2013). In our study, it was found that the functional brain networks of the three groups still had small-world attributes. Additionally, no significant difference existed in global attribute indicators such as Lp, Gamma, Lambda, Sigma, and Eg among the NCs, and LLD baseline and follow-up groups, which was similar to the results of previous studies (Lim et al., 2013; Ajilore et al., 2014).

However, the Cc value was lower in the LLD baseline group than in the NCs group, which suggested that the internal characteristics of the functional brain networks in patients with LLD had been modified. The decrease in Cc value may indicate that the functional division of labor among brain modules was underdeveloped and the order of information processing in the functional brain networks was disordered. Combining recent neuroimaging studies, it can be observed that both the functional and structural brain networks confirm that LLD is a disease of overall brain disconnection (Li et al., 2015; Mak et al., 2016). In LLD baseline group,

Pearson test revealed that the Cc values of functional brain networks were negatively correlated with the scores of HAMD-17, which was similar to the findings of a previous study involving white-matter functional topology features in the MDD population (Li et al., 2020). It may suggested that the Cc value of small-worldness in functional brain networks that has been found perhaps be indicative of ‘trait' markers that are often linked to clinical state. Future research may examine whether using the brain connectome's small-world topology makes it easier to identify LLD and further provides early indications for psychiatric treatment.

A previous cross-sectional study found that the topological properties of the brain functional networks in LLD patients during the remitted period did not recover with the improvement of depressive symptoms, primarily manifested as an increase in Lp length and a decrease in Eg (Wang et al., 2016). In this study, after 6 months of depression treatment, the LLD follow-up group had no significant changes in global indicators such as Cc, Lp, Gamma, Lambda, Sigma, and Eg compared with the LLD baseline group, which may indicate that the disorder of integration and division of labor in brain functional networks may be a characteristic change. In LLD follow-up group, Pearson test found that Lp values of functional brain networks were adversely linked with the RBANS scores. Lp represents the network's overall functional integration and global information transmission capabilities. The lower the functional integration and information transmission, the greater the cognitive impairment, which remains consistent with the previous findings of Xiang et al. (2013). In this study, the small sample size would result in low statistical power of altered small-world topology and RBANS subtests, most of them did not survive after Bonferroni correction. Future research should include more replication samples to better reveal the information flow characteristics of functional brain networks behind cognitive impairment in LLD.

In terms of Ne, compared with the NCs group, the Ne values of bilateral Frontal_Mid_Orb decreased, and the Ne value of Temporal_Pole_Mid_R increased in the LLD baseline group; the Ne values of bilateral Frontal_Mid_Orb and part of cerebellar regions decreased in LLD follow-up group after 6 months of antidepressant therapy compared with the LLD baseline group. The orbitofrontal cortex is primarily involved in information processing, executive control, and negative emotion regulation (Drevets, 2007). A cross-sectional study using diffusion tensor imaging found that the integrity of orbitofrontal nerve fiber bundles was reduced in the first-episode, drug-naive depressed patients (Ma et al., 2007). Our study showed that after 6 months of antidepressant therapy, the attention and visuospatial/constructional scores of the LLD follow-up group did not improve compared with the LLD baseline group, combined with the persistent decrease of Ne values in the orbitofrontal cortex, which may indicated the persistent non-relief of cognitive function, especially executive function, may related to the decrease of Ne values in the orbitofrontal cortex.

Compared to the NCs group, the LLD baseline group had a higher Ne value in the Temporal_Pole_Mid_R, which is thought to belong to the default mode network (DMN) and is primarily associated with self-reflection and automated thinking (Chen et al., 2016). In the LLD baseline group, the increased Ne value of the Temporal_Pole_Mid_R may indicate that it was influenced by negative self-concept and attentional bias. The activation pattern of the frontotemporal network in the LLD baseline group differed from that of the NCs group when causal attribution was made. The middle temporal gyrus may play a defensive role and exhibit abnormal activation (Seidel et al., 2012). Few studies have focused on the role of cerebellar function in the occurrence and development of LLD. In recent years, topological studies of cerebellar function have demonstrated that the cerebellum is involved in the regulation of various functional networks, including cognition, emotion, reward, and autonomic nervous processes, which may be linked to higher cognitive regions of the brain through feedback projections from the thalamus (Schmahmann, 2019). A previous study found that both rLLD and amnestic MCI had changes in functional connectivity between the right middle temporal cortex and the posterior cerebellum and that these changes were negatively correlated with the Mattis Dementia Rating Scale score (Chen et al., 2016). The authors hypothesized that rLLD and amnestic MCI might share similar pathological mechanisms, representing a disease progression continuum to AD. It is worth noting that the cerebellum is structurally divided into anterior and posterior lobes, as well as follicular nodules. There have been a few studies on the roles of various cerebellar subregions in the occurrence and progression of LLD, which should be investigated further.

In terms of DC, the LLD baseline group had lower DC values for bilateral Frontal_Mid_Orb, cerebelum_Crus1_L, cerebelum_9_R, and Vemis_7 compared to the NCs group. In the LLD baseline group, the DC values of Rolandic_Oper_R, Heschl_R, Temporal_Mid_R, and Temporal_Pole_Mid_R increased. A previous study discovered that many older individuals with decreased cognitive function had increased medial temporal lobe activity when compared to the control group, possibly indicating compensatory activation of nerve reserve in the early stages of dementia (Sperling et al., 2008). The elevated DC brain regions were on the right side of the brain, which may be related to the dominant hemisphere theory. It has been demonstrated that the processing of neutral or positive information is primarily controlled by the right hemisphere in patients with MDD, whereas the processing of negative emotional information is primarily controlled by the left hemisphere (Gur et al., 1992). Participants should close their eyes and relax while undergoing rs-fMRI. The altered brain regions with elevated DC in the LLD baseline group were located on the right side of the brain, which may be close to the neutral environment. Despite significant improvement in depressive symptoms, the DC values of cognitive control network (CCN) (such as Frontal_Sup_L and bilateral Frontal_Mid_Orb) and part of the cerebellum in the LLD follow-up group remained lower than in the LLD baseline group.

Compared with the LLD baseline group, the DC values of bilateral insular, Cingulum_Mid_L, Putamen_R, and Heschl_R increased in the LLD follow-up group. Brain regions such as the insular lobe, lateral cingulate, and putamen belong to the salience network (SN), which is closely related to visceral sensation, movement, and emotional experiences. SN can integrate all kinds of emotional experiences to integrate external and internal stimulation information into a subjective sensory state (Singer et al., 2009). After 6 months of treatment using antidepressants, the relief of depressive symptoms may assist in enhancing the perception of these nodal brain regions in SN to the properties of stimulating materials and their role in dealing with complex decision-making behaviors.

Our study has some limitations. Firstly, most of the patients with LLD included in our study were outpatients. During the 6-month antidepressant therapy, although the blood concentration was often monitored, some patients still missed and took fewer drugs, which might have interfered with the accuracy of the study results. Secondly, no single drug was used in the course of treatment, 19 cases took escitalopram oxalate, and only eight cases took sertraline hydrochloride, so there was no subgroup analysis was conducted further in this study. Third, because this study only followed patients with LLD and did not follow the NCs group at the end of 6 months, the comparison of the graph theory indices of the functional brain networks between the LLD follow-up group and the NCs group in the same period is unknown; fourth, the small sample size may have an impact on the robustness of the study findings, so we should expand the sample size in the later stage to include multicenter data to better mine the unique brain activity characteristics of LLD.

## Conclusion

The ability to integrate and divide labor of functional brain networks declines in LLD patients and linked with the depression severity. After the relief of depressive symptoms, the small-world attribute of functional brain networks in LLD patients persists. However, the information transmission efficiency and centrality of some brain regions continue to decline over time, perhaps related to their progressive cognitive impairment.

## Data availability statement

The raw data supporting the conclusions of this article will be made available by the authors, without undue reservation.

## Ethics statement

The studies involving human participants were reviewed and approved by the Beijing Anding Hospital Affiliated to Capital Medical University. The patients/participants provided their written informed consent to participate in this study.

## Author contributions

CL, LL, and WP conceived and designed the research protocol. CL completed the data analyses. DZ, SL, and YL assisted with neuropsychological assessment and data processing of rs-fMRI. PM and YR checked the rs-fMRI data and revised the manuscript. XM provided financial support for this study. All authors contributed to the article and approved the submitted version.
