# Supplementary material for: Altered topological properties of functional brain networks in patients with first episode, late-life depression before and after antidepressant treatment
Source: Front Aging Neurosci. 2023 Mar 6;15:1107320. doi: 10.3389/fnagi.2023.1107320 (PMC10025486; doi:10.3389/fnagi.2023.1107320)
Supplement: Supplementary file 1 [file Data_Sheet_1.docx]

Supplementary Material

**Altered Topological Properties of Functional Brain Networks in Patients With First Episode, Late-Life Depression Before and After Antidepressant Treatment**

**Chaomeng Liu ^1, 2, +^, Li Li ^1, 2, +^, Weigang Pan ^1, 2, +^, Dandi Zhu ^1, 2^, Siyuan Lian ^1, 2^, Yi Liu ^1, 2^, Li Ren ^1, 2^, Peixian Mao ^1, 2^, Yanping Ren ^1, 2*^, Xin Ma ^1, 2*^**

*** Correspondence:** Xin Ma, E-mail address: [maxinanding@ccmu.edu.cn;](mailto:lixiaohong_anding@ccmu.edu.cn;) Yanping Ren, E-mail address: [renyanping@ccmu.edu.cn;](mailto:lixiaohong_anding@ccmu.edu.cn;)

**Supplementary Table 1.** Comparisons of depressive symptoms and cognitive function between the LLD baseline group and LLD follow-up group.

| Characteristic | LLD baseline group | LLD follow-up group | *T* values | *P* values |
| --- | --- | --- | --- | --- |
|  | (n = 27) | (n = 27) |  |  |
| HAMD-17 score | 21.11 ± 2.95 | 6.33 ± 4.47 | 16.193 | < 0.001^*^ |
| RBANS total score | 97.48 ± 11.49 | 104.89 ± 12.48 | -5.358 | < 0.001^*^ |
| Immediate memory | 95.52 ± 12.96 | 102.67 ± 14.39 | -3.095 | 0.005^*^ |
| Visuospatial/Constructional | 98.59 ± 10.31 | 100.26 ± 8.36 | -0.971 | 0.341 |
| Language | 96.41 ± 10.89 | 101.52 ± 7.96 | -2.596 | 0.015^*^ |
| Attention | 111.63 ± 12.36 | 112.96 ± 14.11 | -0.815 | 0.423 |
| Delayed memory | 93.93 ± 10.86 | 99.63 ± 13.04 | -3.397 | 0.002^*^ |

**Note:** *the difference is statistically significant (*P* < 0.05)

**Supplementary Table 2.** Comparisons of global attribute indicators of functional brain networks between the three groups from the AAL116 template.

| Global indicators | NCs group | *t* values | *p* values | LLD  baseline group | *T* values | *P* values | LLD  follow-up group | *T* values | *P* values |
| --- | --- | --- | --- | --- | --- | --- | --- | --- | --- |
| Cc | 0.100 ± 0.011 | 2.676 | 0.010 | 0.091 ± 0.013 | -0.289 | 0.775 | 0.092 ± 0.014 | 2.335 | 0.023 |
| Lp | 1.109 ± 0.097 | -0.356 | 0.723 | 1.120 ± 0.128 | -0.129 | 0.899 | 1.123 ± 0.128 | 0.453 | 0.653 |
| Gamma | 0.917 ± 0.091 | 0.832 | 0.409 | 0.895 ± 0.103 | 1.000 | 0.327 | 0.866 ± 0.111 | 1.846 | 0.071 |
| Lambda | 0.517 ± 0.025 | -1.733 | 0.089 | 0.529 ± 0.025 | -0.030 | 0.977 | 0.529 ± 0.030 | 1.604 | 0.115 |
| Sigma | 0.731 ± 0.068 | 0.878 | 0.384 | 0.713 ± 0.082 | 0.911 | 0.370 | 0.692 ± 0.084 | 1.875 | 0.066 |
| Eg | 0.187 ± 0.017 | 0.000 | > 0.999 | 0.187 ± 0.025 | 0.155 | 0.878 | 0.186 ± 0.022 | 0.187 | 0.853 |

**Note:** The *t* values of the third, sixth, and ninth columns are the results of comparing NCs group and LLD baseline group, LLD baseline group and LLD follow-up group, LLD follow-up group and NCs group, respectively. Additionally, there is a 1:1 correspondence between the values of *t* and *p*. In the comparisons of global attribute indicators, the areas under the curves (AUC) enclosed by the values of each sparsity are generally used.

**Supplementary Table 3.** Comparisons of global attribute indicators of functional brain networks between the three groups from the Dosenbach 160 template.

| Global indicators | NCs group | *T* values | *P* values | LLD  baseline group | *T* values | *P* values | LLD  follow-up group | *T* values | *P* values |
| --- | --- | --- | --- | --- | --- | --- | --- | --- | --- |
| Cc | 0.095 ± 0.015 | 2.235 | 0.030 | 0.085 ± 0.017 | -0.032 | 0.974 | 0.085 ± 0.015 | 2.313 | 0.025 |
| Lp | 1.241 ± 0.122 | 0.481 | 0.633 | 1.227 ± 0.101 | -0.766 | 0.451 | 1.245 ± 0.137 | -0.098 | 0.923 |
| Gamma | 0.841 ± 0.099 | -0.734 | 0.466 | 0.860 ± 0.089 | 1.113 | 0.201 | 0.843 ± 0.097 | -0.094 | 0.925 |
| Lambda | 0.540 ± 0.019 | 0.453 | 0.653 | 0.538 ± 0.015 | -0.197 | 0.845 | 0.538 ± 0.013 | 0.328 | 0.744 |
| Sigma | 0.692 ± 0.080 | -0.885 | 0.380 | 0.710 ± 0.072 | 1.096 | 0.283 | 0.696 ± 0.077 | -0.194 | 0.847 |
| Eg | 0.167 ± 0.018 | -0.303 | 0.763 | 0.168± 0.015 | 0.418 | 0.680 | 0.166 ± 0.020 | 0.036 | 0.972 |

**Note:** The same as Supplementary Table 2.


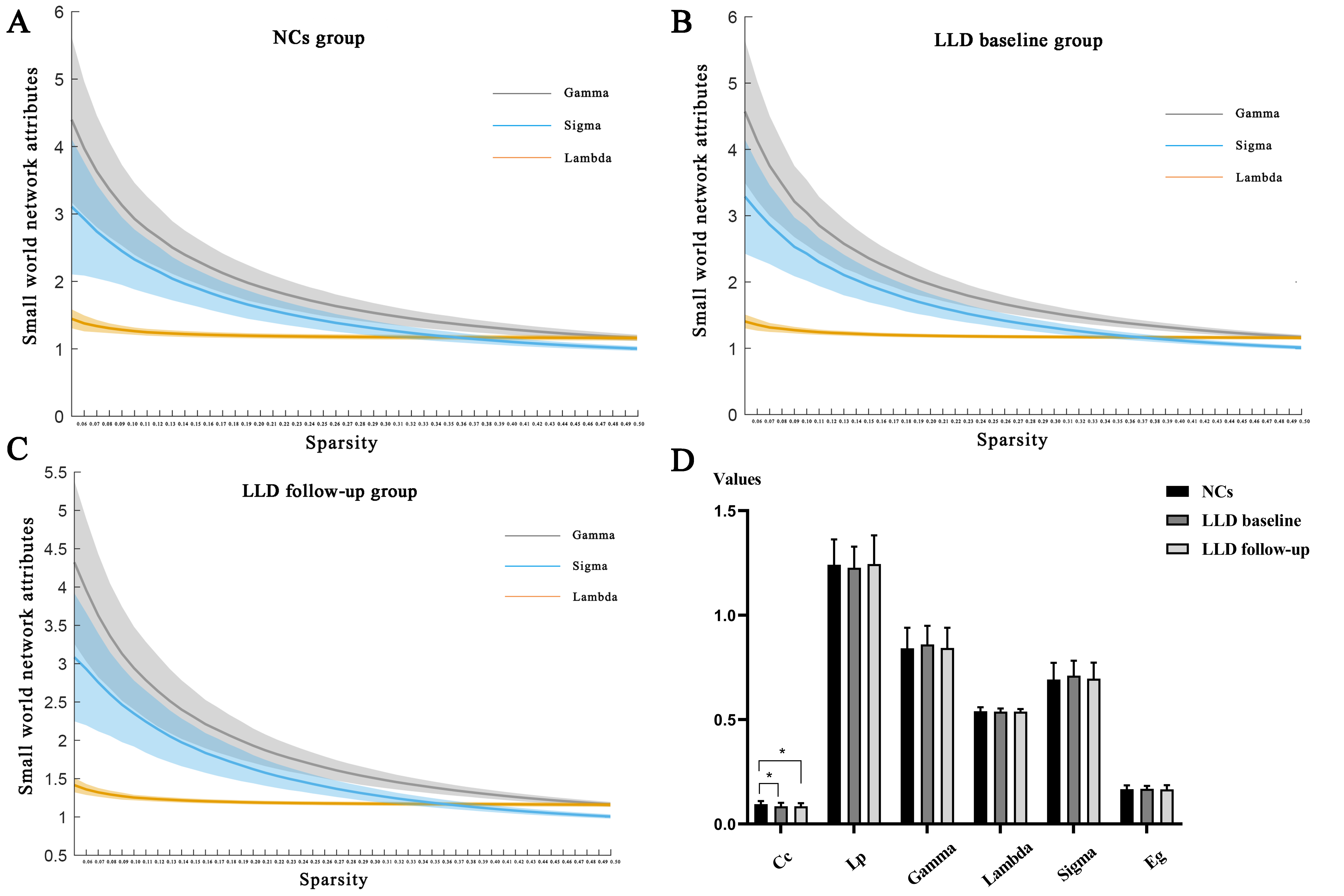


**Supplementary Figure 1.** Comparisons of global indicators of functional brain networks from the Dosenbach 160 template. (A) Small-world attribute indices of functional brain networks along with sparsity in the NCs group; (B) Small-world attribute indices of functional brain networks along with sparsity in the LLD baseline group; (C) Small-world attribute indices of functional brain networks along with sparsity in the LLD follow-up group; (D) Global attribute indicators of functional brain networks in LLD baseline group, LLD follow-up group and NCs group.
